# An Acquired Form of Dandy-Walker Malformation with Enveloping Hemosiderin Deposits

**DOI:** 10.1155/2017/3861608

**Published:** 2017-10-25

**Authors:** Tadashi Shiohama, Ryo Ando, Katsunori Fujii, Hiroki Mukai, Yuki Naruke, Katsuo Sugita, Eiji Kato, Naoki Shimojo

**Affiliations:** ^1^Department of Pediatrics, Graduate School of Medicine, Chiba University, Chiba, Japan; ^2^Division of Pediatric Neurosurgery, Chiba Children's Hospital, Chiba, Japan; ^3^Department of Radiology, Chiba University Hospital, Chiba, Japan; ^4^Division of Diagnostic Pathology, Chiba Children's Hospital, Chiba, Japan; ^5^Division of Child Health, Faculty of Education, Chiba University, Chiba, Japan; ^6^Division of Neonatology, Funabashi Central Hospital, Chiba, Japan

## Abstract

Dandy-Walker malformation (DWM) is a posterior fossa anomaly characterized by hypoplasia and upward rotation of the cerebellar vermis and cystic dilation of the fourth ventricle. The cyst of DWM rarely extends posteriorly to almost completely fill the entire posterior fossa, which mimics primary cerebellar agenesis, a cerebellar porencephalic cyst, and an arachnoid cyst due to the lack of clarity of the thin cystic wall. A 10-month-old female born at 23 weeks' gestation with cerebellar hemorrhage in the neonatal period was admitted to our hospital with dysphagia and side-to-side head bobbing. The detection of hemosiderin deposits enveloping the cyst wall by T2 star-weighted angiography (SWAN) was useful for the differential diagnosis of an acquired form of DWM from primary cerebellar agenesis. Cyst fenestration successfully improved dysphagia and head bobbing. A pathological specimen of the perforated cyst consisted of collagen fibers with hemosiderin deposits but lacked congenital cyst components. In infants with posterior fossa cysts, SWAN will be useful for a differential diagnosis between DWM and primary cerebellar agenesis.

## 1. Introduction

Dandy-Walker malformation (DWM) is a posterior fossa anomaly characterized by hypoplasia and upward rotation of the cerebellar vermis and cystic dilation of the fourth ventricle. DWM was previously recognized as a congenital brain malformation but is rarely caused by intrauterine and neonatal brain insults including cerebellar hemorrhage and intraventricular hemorrhage [1–3] and, thus, has been named “acquired” DWM [1]. The cyst of DWM rarely extends posteriorly to almost completely fill the entire posterior fossa, which mimics primary cerebellar agenesis [4], a cerebellar porencephalic cyst [5], and an arachnoid cyst [6] due to the lack of clarity of the thin cystic wall; however, an accurate differential diagnosis is critical for selecting an appropriate surgical intervention.

We herein describe an infantile case of DWM caused by cerebellar hemorrhage in the neonatal period. Although a nonrecognizable cerebellar hemisphere in the posterior fossa mimicked primary cerebellar agenesis [4], T2 star-weighted angiography (SWAN) successfully detected hemosiderin deposits enveloping the cystic lesion, which led to the diagnosis of an acquired form of DWM and selection of a surgical intervention approach.

## 2. Case Presentation

**Figure 1:**
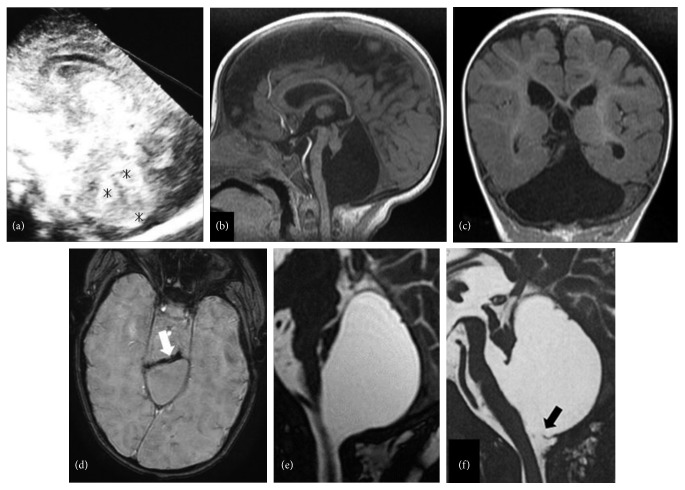
Echography on day 3 showing a high echoic lesion in the cerebellar hemisphere (a, black asterisks). Sagittal (b) and coronal (c) spoiled gradient recalled echo (SPGR) imaging at 10 months old showing a nonrecognizable bilateral cerebellar hemisphere and hypoplastic cerebellar vermis and brainstem. Axial T2 star-weighted angiography (SWAN) showed hemosiderin deposits enveloping the cyst wall (d, white arrow) at presurgery. Midsagittal fast imaging employing steady-state acquisition (FIESTA) did not identify the cyst wall before surgery (e) but detected it after surgery (f, black arrow).

A 10-month-old female was admitted to our hospital with intermittent side-to-side head bobbing that had persisted for one month. She was delivered via cesarean section at 23 weeks' gestation with a birth weight of 575 g due to chorioamnionitis and placental abruption. Apgar scores were 6 and 8 at 1- and 5-minute postpartum. A brain echogram on day 3 revealed high echoic lesions within the cerebellar hemispheres, suggesting the presence of acute cerebellar hemorrhage. Neither a cerebral abnormal lesion nor intraventricular hemorrhage was detected (Figure 1()). She was discharged after 4 months without motor or feeding dysfunctions. Auditory brainstem responses showed no abnormal findings.

At 10 months old (5 months' corrected age), she controlled her head and was rolling over, but intermittently presented side-to-side head bobbing regardless of her posture, particularly in the morning. A physical examination showed left intermittent exotropia and mild truncal instability, but not nystagmus, involuntary movements, or an apparent pyramidal sign. Her weight was 6.2 kg (−1.0 standard deviation (SD)), height 58.8 cm (−1.7 SD), and head circumference 41.3 cm (0 SD) of the Japanese corrected age-matched references. The clinical symptoms of increased intracranial pressure such as seizures, colic, and vomiting were absent. Electroencephalography showed no epileptic discharge including hypsarrhythmia.

Brain MRI at 10 months old revealed an almost nonrecognizable cerebellum and a giant CSF cyst filling the posterior fossa with enveloping hemosiderin deposits and brainstem compression (Figures 1()). Fast imaging employing steady-state acquisition (FIESTA) did not reveal the presence of the cyst wall (Figure 1()).

Cyst fenestration with a posterior cervical approach was performed to improve brainstem compression at 12 months old. Neuroendoscopic observations from the perforated hole reconfirmed that the posterior fossa cyst was an individual cyst without a septum. After surgery, dysphagia immediately improved, and head bobbing disappeared within one month. Postsurgical FIESTA images detected the cyst wall segment (Figure 1()).

**Figure 2:**
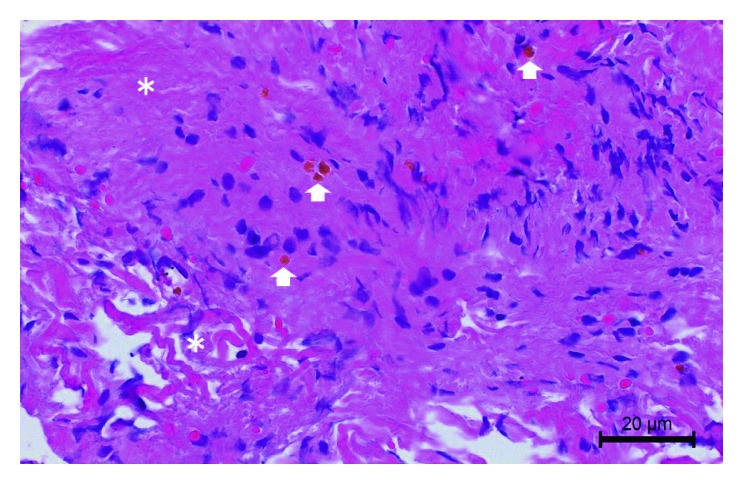
A histological examination of the perforated cyst segment showed collagen fibers (asterisk), spindle cells, and hemosiderin deposits (white arrows) (hematoxylin and eosin staining, bar: 20 µm). A section of the cyst wall was fixed with formalin and embedded in paraffin.

A histological examination of a perforated cyst segment showed collagen fibers with hemosiderin deposits (Figure 2()). An immunohistological examination revealed that the specimen lacked arachnoid and ependymal cells (recognized as epithelial membrane antigen- (EMA-) positive cells) as well as glia cells (recognized as glial fibrillary acidic protein- (GFAP-) positive cells).

## 3. Discussion

We herein presented an infantile case of an acquired form of DWM associated with cerebellar hemorrhage in the neonatal period, which ultimately compressed the brainstem in the late infantile period. In this clinical course, side-to-side head bobbing was noted as an initial symptom before dysphagia and the detection of brainstem compression and hydrocephalus on neuroimaging. This nonvoluntary movement was previously reported as the “no-no” type bobble-head doll sign, which has been recognized as voluntary rhythmic head waving in an attempt to release the cerebrospinal fluid obstruction caused by the posterior fossa cyst [7].

The brain morphology of this case was characterized as a subtotal absence of the cerebellar vermis and hemispheres, a hypoplastic brainstem, and a giant cyst filling the posterior fossa and fourth ventricle. Regarding cyst distribution, a neuroendoscopic view at cyst fenestration showed a single cyst extending from the posterior fossa to the fourth ventricle. Furthermore, brain MRI at 2 days post surgery showed air distribution in the lateral ventricles. These results indicated communication between the lateral ventricles and posterior fossa cyst. Collectively, neuroimaging and neuroendoscopic findings resulted in a diagnosis of an acquired form of DWM. The posterior fossa cyst of “acquired” DWM grows after birth [1]; therefore, the absence of an enlarged posterior fossa in this case did not reject the diagnosis of an acquired form of DWM.

In terms of a histological diagnosis, DWM generally contains a glial layer covered on the lateral surface of the cyst by an ependymal layer [6]. The cyst wall specimen of this case consisted of connective tissue with hemosiderin deposits but lacked congenital cyst components such as arachnoid, ependymal, choroid, and glial cells. This discrepancy in histological findings may be because the specimen was obtained from the penetration site only.

In the pathogenesis in an acquired form of DWM, cerebellar hemorrhage in the neonatal period may have played a role by obstructing the outlet foramina of the fourth ventricle. Cerebellar hemorrhage rarely occurs in premature infants born before 25 weeks' gestation [8, 9] because the cerebellar germinal matrix within the external layer, which is the typical origin of cerebellar hemorrhage, transiently develops from 24 weeks' gestation. Importantly, cerebellar hemorrhage in the neonatal period often does not induce any clinical symptoms and is easily overlooked with echography [10]. If cerebellar hemorrhage is overlooked, the differential diagnosis of a posterior fossa cyst depends heavily on neuroimaging studies.

The cyst wall of the posterior fossa cyst in this case was not detected by SPGR or FIESTA, whereas SWAN revealed hemosiderin deposits enveloping the posterior fossa cyst. MR cisternography including FIESTA is useful in evaluations of the cystic wall because of the higher contrast-to-noise ratio and brain tissue/CSF contrast [11] but lacks contrast between soft tissues and even between soft tissues and bone [11]. SWAN was more sensitive than FIESTA for revealing the cyst wall in this case, albeit indirectly, by detecting hemosiderin deposits on the cyst wall, and this may have been because the cyst wall was in close contact with the arachnoid membrane.

In conclusion, we herein present a case of an acquired form of DWM associated with cerebral hemorrhage in the neonatal period. The detection of hemosiderin deposits enveloping the cyst wall was useful for a differential diagnosis of “acquired” DWM from primary cerebellar agenesis.
